# Hidden Infections, Emerging Resistance: Underdiagnosed *Plasmodium vivax* and Multidrug Resistance-1 (*pvmdr1*) Gene Amplification in the Guiana Shield

**DOI:** 10.1093/infdis/jiag196

**Published:** 2026-04-08

**Authors:** Maria Carolina Silva de Barros Puça, Maria Eduarda Pereira Mascarenhas, Queralt Bullich Huguet, Yanka Evellyn Alves Rodrigues Salazar, Iuri Rodrigues Nogueira, Jaime Louzada, Anielle de Pina-Costa, André Machado de Siqueira, Michelle de Oliveira e Silva, Dhélio Batista Pereira, Simone Ladeia-Andrade, Marcelo Urbano Ferreira, Joseli Oliveira-Ferreira, José Pedro Gil, Tais Nobrega de Sousa

**Affiliations:** Molecular Biology and Malaria Immunology Research Group, Instituto René Rachou, Fundação Oswaldo Cruz (FIOCRUZ), Belo Horizonte, Minas Gerais, Brazil; Department of Microbiology, Tumor and Cell Biology, Karolinska Institutet, Solna, Sweden; Molecular Biology and Malaria Immunology Research Group, Instituto René Rachou, Fundação Oswaldo Cruz (FIOCRUZ), Belo Horizonte, Minas Gerais, Brazil; Department of Microbiology, Tumor and Cell Biology, Karolinska Institutet, Solna, Sweden; Molecular Biology and Malaria Immunology Research Group, Instituto René Rachou, Fundação Oswaldo Cruz (FIOCRUZ), Belo Horizonte, Minas Gerais, Brazil; Department of Microbiology, Tumor and Cell Biology, Karolinska Institutet, Solna, Sweden; Universidade Federal de Roraima, Boa Vista, Roraima, Brazil; Universidade Federal de Roraima, Boa Vista, Roraima, Brazil; Universidade Federal Fluminence, Rio de Janeiro, Brazil; Instituto Nacional de Infectologia Evandro Chagas, Fundação Oswaldo Cruz (FIOCRUZ), Rio de Janeiro, Brazil; Centro de Pesquisa em Medicina Tropical de Rondônia—CEPEM, Porto Velho, Rondônia, Brazil; Centro de Pesquisa em Medicina Tropical de Rondônia—CEPEM, Porto Velho, Rondônia, Brazil; Oswaldo Cruz Institute, Fundação Oswaldo Cruz, Rio de Janeiro, Brazil; Department of Parasitology, Institute of Biomedical Sciences, Universidade de São Paulo (USP), São Paulo, Brazil; Global Health and Tropical Medicine (GHTM), Institute of Hygiene and Tropical, NOVA University of Lisbon, Lisbon, Portugal; Oswaldo Cruz Institute, Fundação Oswaldo Cruz, Rio de Janeiro, Brazil; Department of Microbiology, Tumor and Cell Biology, Karolinska Institutet, Solna, Sweden; Global Health and Tropical Medicine (GHTM), Institute of Hygiene and Tropical, NOVA University of Lisbon, Lisbon, Portugal; Molecular Biology and Malaria Immunology Research Group, Instituto René Rachou, Fundação Oswaldo Cruz (FIOCRUZ), Belo Horizonte, Minas Gerais, Brazil; Department of Microbiology, Tumor and Cell Biology, Karolinska Institutet, Solna, Sweden

**Keywords:** malaria, *Plasmodium mixed infection*, molecular diagnosis, *pvmdr1 copy number variation*, drug resistance

## Abstract

**Background:**

*Plasmodium vivax* is the most widespread malaria parasite, and its cocirculation with *Plasmodium falciparum* often leads to underdiagnosis and underestimated transmission. We investigated whether underdiagnosis contributes to the selection and spread of drug-resistant parasites in coendemic areas of the Brazilian Amazon.

**Methods:**

We enrolled 727 confirmed malaria cases from three Brazilian states, primarily from Roraima in the Guiana Shield, a region characterized by cocirculation of *P. vivax* and *P. falciparum*, intense cross-border mobility, illegal mining activities, and informal antimalarial drug use. To examine indirect *P. vivax* exposure to antimalarial targeting *P. falciparum*, we evaluated *pvmdr1* copy number variation (CNV).

**Results:**

In Roraima, molecular diagnosis revealed frequent misclassification of mixed infections, with *P. vivax* often missed by microscopy due to higher *P. falciparum* densities, demonstrating the limitations of microscopy in accurately detecting mixed infections. The *pvmdr1* CNV exhibited significant geographic variation. While nearly all isolates from the western Amazon carried a single copy, *pvmdr1* amplification was detected in 60% of Roraima isolates. In addition to this spatial heterogeneity, temporal analysis revealed significant shifts in amplification patterns over the years, rising to over 70% after 2020.

**Conclusions:**

These findings emphasize the critical importance of accurate diagnosis and rational use of antimalarials, as inadequate or unintended drug exposure may inadvertently drive the selection and spread of parasite strains with reduced drug susceptibility.

## Teaser

Underdiagnosis of *Plasmodium vivax* and frequent *pvmdr1* amplifications in border regions of the Brazilian Amazon highlight diagnostic gaps, drug selective pressures, and the potential emergence of resistant parasite populations threatening malaria control.

*Plasmodium vivax* is the most widespread human malaria parasite, responsible for most cases outside Africa and representing a major public health concern in Asia, Oceania, and the Americas [1]. Although often considered less severe than *Plasmodium falciparum*, *P. vivax* malaria causes substantial morbidity, recurrent episodes, and has been increasingly associated with severe outcomes [2, 3]. Its biological particularities and emerging drug resistance remain key obstacles to malaria elimination.

In the Brazilian Amazon, malaria transmission occurs in rural areas, Indigenous communities, gold mining sites, and urban areas [4, 5]. Among Indigenous populations, the Yanomami people, who inhabit a vast territory spanning Brazil and Venezuela, are disproportionately affected, facing high malaria incidence due to limited healthcare access, intense cross-border mobility, and illegal mining activities within their lands [6, 7]. Between 2016 and 2023, annual malaria cases in Brazil fluctuated between 130,000 and 190,000, with most infections caused by *P. vivax* [5]. However, surveillance data indicate a recent rise in *P. falciparum* infections, especially in border states such as Roraima, where imported cases and population mobility contribute to transmission [8–10]. The coexistence of both species raises concerns about mixed infections and *P. vivax* underdiagnosis, due to its typically low parasitemia and hidden reservoirs in the spleen and bone marrow [11].

The frontline treatment for *P. vivax* infections in Brazil is a combination of chloroquine (CQ) and primaquine (PQ), where CQ eliminates blood-stage parasites and PQ achieves radical cure by targeting dormant liver stages [12]. While CQ-resistant *P. vivax* strains were first reported in Papua New Guinea and Indonesia, subsequent reports from multiple endemic areas have raised global concern [13, 14]. Unlike *P. falciparum*, for which resistance mechanisms are relatively well characterized, the molecular basis of CQ resistance in *P. vivax* remains poorly understood. Orthologs of known *P. falciparum* resistance genes, such as the *P. vivax* CQ resistance transporter ortholog gene (*pvcrt-o*) and the *P. vivax* multidrug resistance-1 (*pvmdr1*), have been the primary focus of molecular surveillance [15].

Amplification of the *pvmdr1* gene has been associated with increased parasite sensitivity to CQ, alongside reduced susceptibility to mefloquine (MQ) [16]. Amplification of *pvmdr1* has been rarely detected in the Americas and Africa (1%–3%) [17], but higher frequencies (up to 59%) have been reported in French Guiana, where MQ was extensively used, and *P. vivax* was likely under indirect drug pressure [18, 19]. In Asia, *pvmdr1* amplification ranges from 4%–33% in Cambodia and 7%–39% in Thailand [19–21]. In Brazil, *pvmdr1* amplification has been described at low to moderate frequencies, ranging from 1% to 46% across different states [22–24], with the highest estimate based on a small sample set (Supplementary Table 1). Although MQ is not commonly used to treat *P. vivax*, indirect drug pressure may occur in areas where *P. vivax* and *P. falciparum* coexist, particularly in regions where MQ has been administered as monotherapy or as part of artemisinin-based combination therapy for *P. falciparum* malaria [19, 25].

Evidence from Africa and Southeast Asia shows that artemether–lumefantrine (AL) treatment selects *P. falciparum* strains with increased *pfmdr1* copy numbers [26]. This observation is particularly relevant for Brazil, where AL is the first-line therapy for uncomplicated *P. falciparum* malaria and is also recommended, along with PQ, for the treatment of *P. vivax* recurrences [12]. Such a scenario might provide a selective landscape that indirectly favors the emergence and spread of *P. vivax* strains carrying multiple copies of *pvmdr1*.

In this context, an important question is whether underdiagnosis of *P. vivax* contributes to the selection and spread of drug-resistant parasites in areas where *P. falciparum* and *P. vivax* cocirculate. To address it, we conducted molecular diagnosis and assessed *pvmdr1* CNV in *P. vivax* isolates collected over 8 years from three epidemiologically distinct Brazilian sites: Roraima, characterized by high mobility, mining, mixed infections, and informal drug use, and Acre and Rondônia States, where mixed infections are less prevalent. We aimed to investigate *pvmdr1* amplification patterns and their implications for malaria treatment in the Amazon.

## METHODS

### Study Area

**Figure 1.**
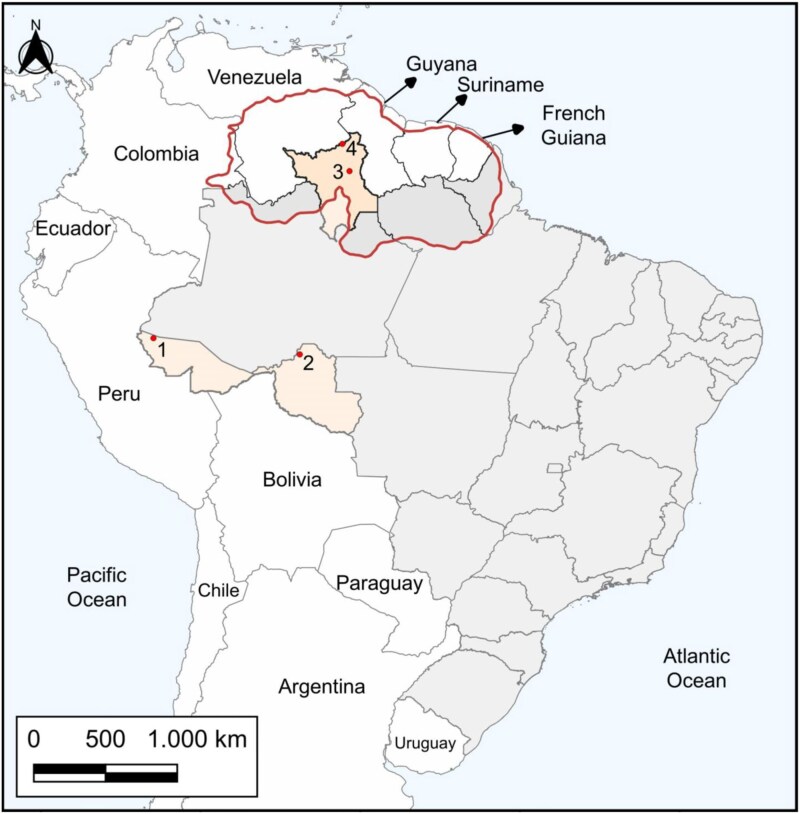
The map shows the extent of the Guiana Shield in northern South America, as delineated in the figure. The panel highlights the 4 study sites in the Brazilian Amazon: (1) Mâncio Lima (Acre), (2) Porto Velho (Rondônia), (3) Boa Vista (Roraima), and (4) Pacaraima (Roraima). Acre and Rondônia are located in the western Amazon, bordering Peru and Bolivia, while Roraima lies in the northern Amazon, sharing borders with Venezuela and Guyana.

The study was conducted in three sites across the Brazilian Amazon: Roraima, Acre, and Rondônia States. These regions differ in malaria transmission dynamics but remain highly endemic for *P. vivax* and, in some areas, *P. falciparum* [5]. Acre and Rondônia are located in the western Amazon, bordering Peru and Bolivia, whereas Roraima lies in the central northern Amazon, sharing borders with Venezuela and Guyana (Figure 1).

Distinct epidemiological contexts were observed across the 3 study sites during the periods evaluated. Roraima presented a malaria scenario marked by rising incidence since 2017, a substantial contribution from imported cases from neighboring countries, and the cocirculation of *P. vivax* and *P. falciparum*, particularly in the municipalities of Boa Vista and Pacaraima. In contrast, malaria transmission in Acre and Rondônia was comparatively more stable and predominantly driven by local transmission, with *P. vivax* accounting for most reported cases and mixed infections remaining rare. Additional epidemiological details are provided in the Supplementary material.

### Study Population

Patients with laboratory-confirmed malaria, as determined by microscopy of Giemsa-stained thick blood smears, were enrolled at each study site. In Roraima, 538 individuals over 12 years old with symptomatic *P. vivax* (n = 287), *P. falciparum* (n = 213), or mixed infections (n = 38) were recruited at the Cosme e Silva Polyclinic (Boa Vista) and Posto Alaide do Carmo Bruce Fernandes (Pacaraima), between 2016 and 2024. In Acre, patients aged 5–70 years with *P. vivax* mono-infection (n = 84) were enrolled at local government-run clinics between June 2014 and July 2015. Treatment followed national guidelines, with *P. vivax* infections treated with CQ plus PQ (3.5 mg/kg over 7 days) and *P. falciparum* infections treated with AL, combined with a single dose of PQ (0.75 mg/kg). In Rondônia, adult patients with *P. vivax* (n = 105) were recruited at Centro de Pesquisa em Medicina Tropical between March and September 2023. The mean age of participants in Rondônia was 43.2 years (interquartile range [IQR]: 33–54), and most participants were male (75.4% men and 24.6% women). In Acre, the mean age was 26.5 years (IQR: 15–37), with 64.2% men and 35.8% women. In Roraima, the mean age was 34.7 years (IQR: 26–43), with 72% men and 28% women. Full inclusion and exclusion criteria, diagnostic methods, and treatment regimens are described in the Supplementary material.

### Ethical Approvals and Participant Consent

The study was approved by the ethics committees of the René Rachou Institute/Fiocruz (nos. 2.803.756 and 2.243.058), the Institute of Biomedical Sciences, University of São Paulo (1169/CEPSH, 2014), and the Karolinska Institutet (2017/499-32). Written informed consent was obtained from all participants or their legal guardians, and assent was provided by minors under 18 years of age.

### Malaria Molecular Diagnosis and Quantification of Parasitemia

We used 200-μL aliquots of venous blood samples collected from each patient to isolate parasite DNA, using QIAamp DNA blood kits (QIAGEN, Hilden, Germany).

All samples underwent molecular diagnosis of malaria by quantitative real-time polymerase chain reaction (qPCR) targeting multicopy genes in the parasite genome. For the samples from Roraima, molecular diagnosis was based on the amplification of *Pfr364* and *Pvr47* for the detection of *P. falciparum* and *P. vivax*, respectively. The detection threshold was 0.66 copies/μL for *P. vivax* and 3.27 copies/μL for *P. falciparum* [27]. For samples from Acre, molecular diagnosis was conducted using a qPCR assay targeting the 18S rRNA genes of *P. falciparum* and *P. vivax*. Standard curves for each assay indicated a detection threshold of approximately 2 parasites/μL of blood [28]. For samples from Rondônia, molecular diagnosis was performed using the commercial IBMP Biomol Malária kit (Instituto de Biologia Molecular do Paraná-IBMP, Curitiba, Brazil). According to the manufacturer's validation, the limits of detection with 95% confidence (LoD95) are 120 copies/μL for *P. falciparum* and 111 copies/μL *P. vivax*.

Parasitemia of the samples from Roraima was quantified by qPCR using standard curves generated from plasmid DNA containing the target sequences of *P. falciparum* and *P. vivax*, following a previously published protocol [29]. For *P. falciparum*, eleven 5-fold serial dilutions were prepared, ranging from 8.0 × 10^6^ to 8.2 × 10^−1^ copies/μL. For *P. vivax*, 11 dilutions were prepared, ranging from 1.6 × 10^7^ to 1.6 × 10^0^ copies/μL. Quantitative real-time polymerase chain reaction reactions were performed in triplicate. Parasite densities (parasites/μL) were derived from quantified copy numbers (copies/μL) based on previously determined calibration equations, corresponding to 0.5 parasites/µL per 1 copy/µL for *Pvr47* and 1.7 parasites/µL per 1 copy/µL for *Pfr364* [29].

The detailed DNA extraction and qPCR protocols, including primers, probes, reagent concentrations, and cycling conditions, are provided in the Supplementary material.

### Determination of Copy Number Variation of the *pvmdr1* Gene

The copy number variation (CNV) of the *pvmdr1* gene was determined by qPCR using previously described probes and primers, with the *P. vivax* β-tubulin gene used as the reference [23, 25].

Amplifications were run in triplicate, and CNV was estimated using the ΔΔCt method. Plasmids containing 1 or 2 copies of *pvmdr1*, along with a single copy of *pvtubulin* were used as calibrators. Samples were classified as single copy when the relative quantification value ranged from 0.5 to 1.4, and as multiple copies when ≥1.4. Only samples with cycle threshold (Ct) values <35 and a Ct standard deviation <0.5 were included in the analysis.

We defined a relative quantification threshold of 1.4 to classify infections as having multiple copies of the *pvmdr1* gene. This threshold was originally established for *P. falciparum*, where it reliably identified infections containing minority subpopulations with *pfmdr1* gene, corresponding to an estimated minimum of approximately 25% of parasites carrying *pvmdr1* amplification within an infection, as demonstrated in artificial mixture experiments [26]. Further technical details are provided in the Supplementary material.

## RESULTS

### Temporal Variation in *P. Vivax* Malaria Underreporting

**Figure 2.**
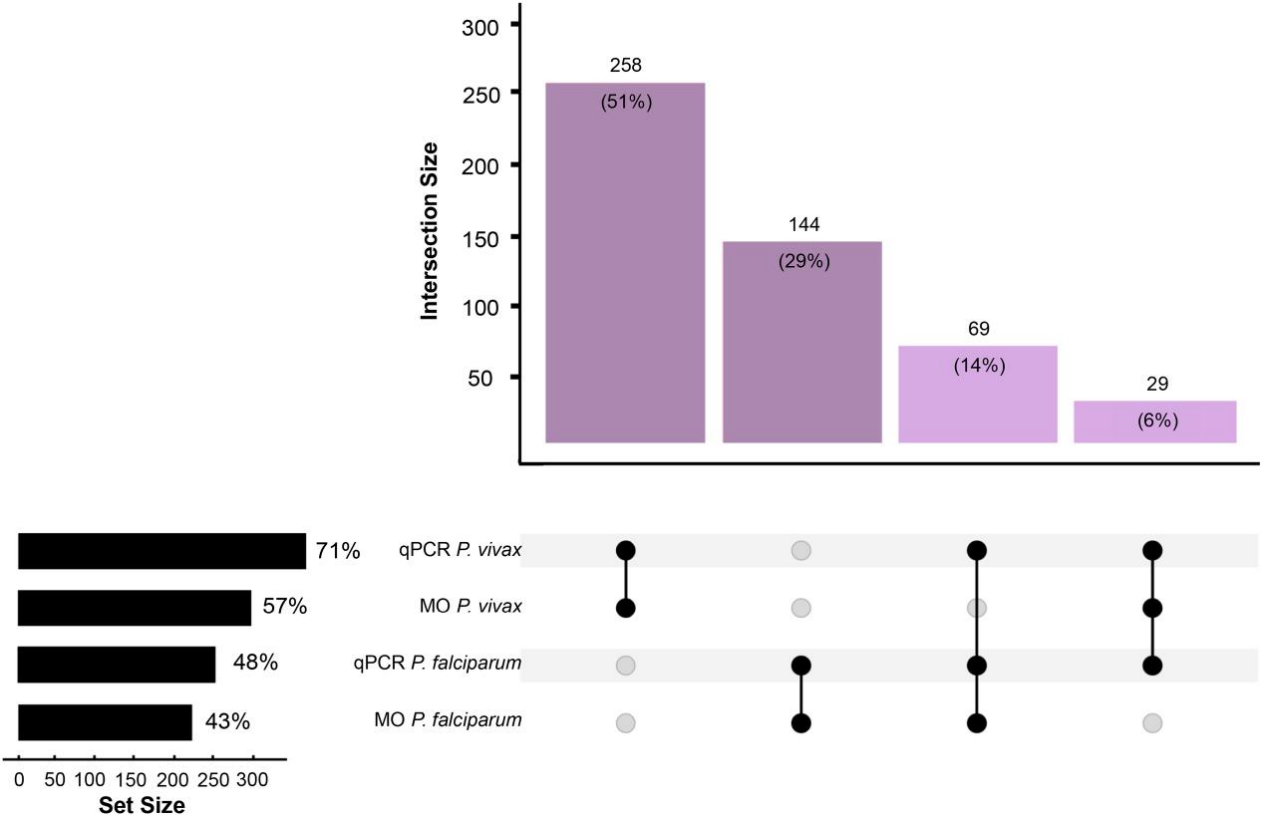
Intersection of *Plasmodium* spp. detection by microscopy (MO) and quantitative polymerase chain reaction (qPCR). The bar plots represent the number of samples detected exclusively by each method or by combinations of techniques. The horizontal set bars indicate the total number of samples detected by each diagnostic method (set size). The vertical intersections show the number of samples detected by the specific combination of methods (intersection size). Bars in darker green indicate samples for which qPCR and microscopy results agreed.

We analyzed a total of 500 samples collected in Roraima that were initially classified as mono-infections by *P. vivax* (*n* = 287, 57.4%) or *P. falciparum* (*n* = 213, 42.6%) based on light microscopy. However, the molecular analyses later identified approximately 20% of these samples (*n* = 98) as mixed infections (*P. falciparum* and *P. vivax*), indicating the concurrent presence of both species. The comparison between diagnostic methods revealed that *P. vivax* is more frequently undetected (*n* = 69) than *P. falciparum* (*n* = 29), indicating a greater potential for silent circulation of this species, especially in mixed infections (Figure 2).

**Figure 3.**
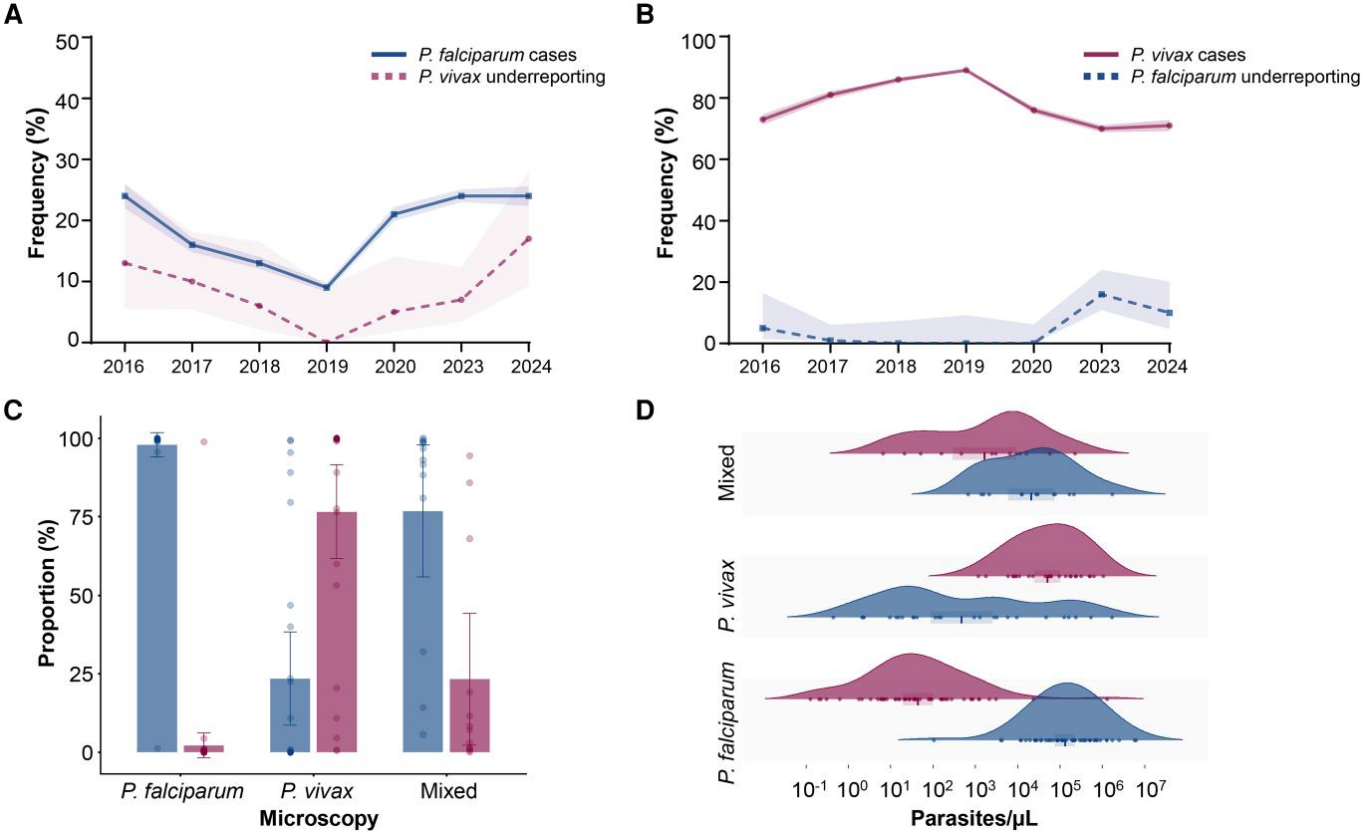
Frequency of *Plasmodium falciparum* and *Plasmodium vivax* cases and species-specific underreporting in Boa Vista-Roraima, Brazil (2016–2024). *A*, *P. falciparum* cases (blue solid line) represent the proportion of microscopy-diagnosed mono-infections recorded in SIVEP-Malaria relative to total malaria cases. *P. vivax* underreporting (red dashed line) corresponds to samples initially classified as *P. falciparum* mono-infections by microscopy but later identified as mixed infections containing *P. vivax* by qPCR. *B*, *P. vivax* cases (red solid line) represent the proportion of microscopy-diagnosed mono-infections in SIVEP-Malaria relative to total malaria cases. *P. falciparum* underreporting (blue dashed line) corresponds to samples initially classified as *P. vivax* mono-infections by microscopy but later identified as mixed infections containing *P. falciparum* by qPCR. For all underreporting estimates, the denominator corresponds to the total number of mono-infection samples analyzed in each specific year. Shaded areas around the lines indicate 95% confidence intervals (95% CIs). *C*, Proportion of *P. falciparum* (blue) and *P. vivax* (pink), based on qPCR-measured pasitemia, according to the species identified by microscopy. *D*, Parasite density distributions for *P. falciparum* and *P. vivax* in single and mixed infection. Vertical bars represent geometric means, and shaded areas indicate 95% CI.

After analysis of the complete dataset, samples were stratified by year of collection, revealing 2 distinct periods: period 1 (2016–2019) and period 2 (2020–2024), which exhibited different profiles of *P. vivax* malaria underreporting as identified by qPCR (Figure 3; Supplementary Tables 2 and 3). In the first period, the proportion of underreported cases decreased from 13% to 0%, whereas in the second period, the opposite trend was observed, with underreported *P. vivax* cases increasing from 5% to 17%. When compared with microscopy-confirmed *P. falciparum* cases from the Brazilian Epidemiological Surveillance System for Malaria (SIVEP-malaria), an association was observed (Figure 3*A*), suggesting that underreported *P. vivax* cases followed the same pattern as reported *P. falciparum* cases. Underreporting of *P. falciparum* cases changed only in the last 2 years, rising from 5% in 2016% to 16% in 2023, and did not follow the overall pattern of *P. vivax* case numbers (Figure 3*B*).

Through the parasite quantification assay, we found that the relative proportions of species in qPCR-detected mixed infections differed markedly, particularly when *P. falciparum* was the predominant species (Figure 3*C*). Consistently, parasite density analysis showed that *P. falciparum* exhibited higher parasite loads than *P. vivax* in both single and mixed infections (Figure 3*D*; Supplementary Table 4), which may partly account for the consistent underdiagnosis of *P. vivax* in mixed infections.

### Analysis of the *pvmdr1* Copy Number

To investigate the potential consequences of indirect and suboptimal antimalarial drug exposure in *P. vivax*, particularly those used against *P. falciparum*, in this coendemic area, we examined the prevalence and distribution of *pvmdr1* CNV in our study population. A total of 400 isolates were analyzed, including 211 out of 287 *P. vivax* mono-infections from Roraima. In addition, we compared the *pvmdr1* CNV pattern with 189 samples from the western Amazon (84 from Acre State and 105 from Rondônia State).

**Figure 4.**
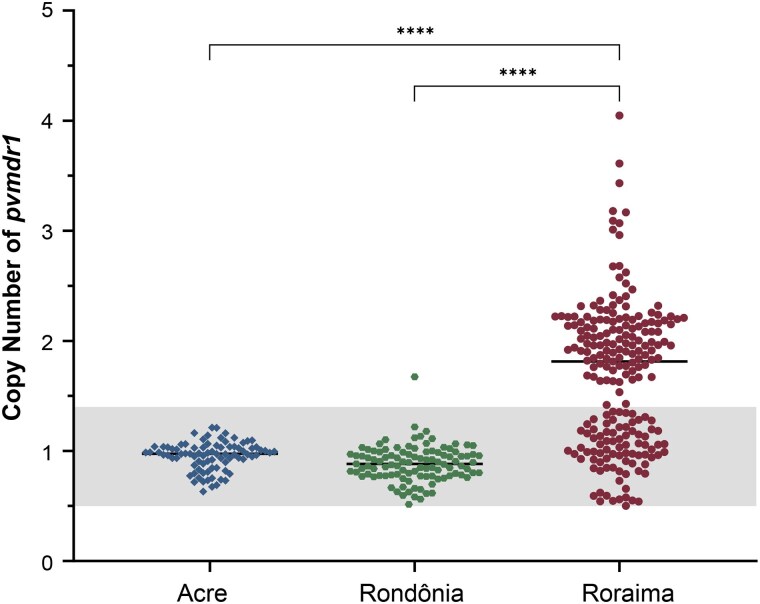
Copy number variation of the *pvmdr1* gene in *Plasmodium vivax* isolates from 3 states of the Brazilian Amazon. Each point represents an individual isolate, and the shaded area marks the cutoff range for single-copy isolates. Kruskal–Wallis test was used to compare groups.

In Roraima, a distinct scenario emerged, with 60% (n = 127) of the isolates carrying multiple copies of the *pvmdr1* gene. The quantification values varied widely (1–4 copies), suggesting marked intrapopulation diversity. In contrast, all isolates analyzed in Acre presented only 1 copy of *pvmdr1*, a pattern similar to that observed in Rondônia, where 99% (104/105) also carried a single copy (Figure 4). This geographic heterogeneity underscores both the higher frequency of *pvmdr1* amplification in Roraima and the marked differences among areas within the Amazon region.

**Figure 5.**
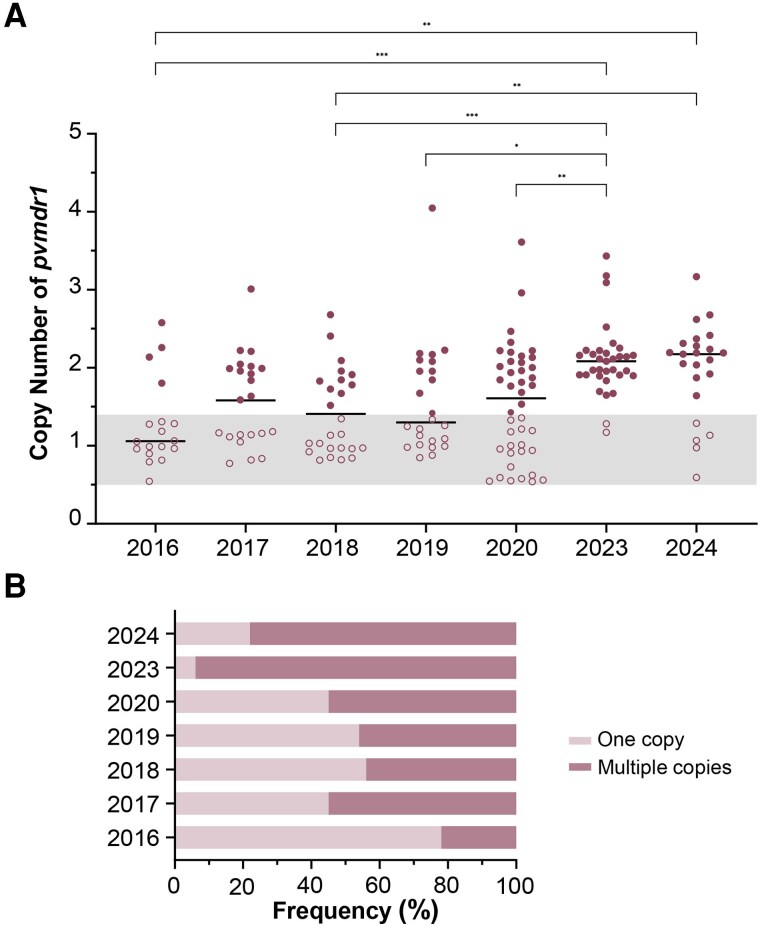
Temporal dynamics of *pvmdr1* copy number variation in *Plasmodium vivax* isolates from Roraima, Brazil. *A*, Distribution of *pvmdr1* copy number per year from 2016 to 2024. Each point represents an individual isolate, and the horizontal lines indicate the median values, filled circles indicate isolates with multiple copies of *pvmdr1* (relative quantification >1.4), and open circles indicate isolates with a single copy (relative quantification between 0.5 and 1.4). The shaded area marks the cutoff range for single-copy isolates. Kruskal–Wallis test was used to compare groups. *B*, Annual frequency of *pvmdr1* CNV.

In addition to this spatial variation, the temporal analysis also revealed significant changes in the *pvmdr1* amplification pattern in Roraima over the years (Figure 5*A*). The median copy number increased progressively from 2020, with statistically significant differences compared with previous years (2016–2019). This trend is further supported by the analysis of isolates grouped by *pvmdr1* copy number, which shows a reversal in distribution: while single-copy isolates generally predominated before 2020, multiple-copy isolates became dominant from 2020 onward, reaching over 70% in 2023–2024 (Figure 5*B*). Together, these findings reveal striking geographic and temporal heterogeneity in *pvmdr1* CNV, with Roraima emerging as a hotspot of gene amplification in *P. vivax* populations of the Brazilian Amazon.

## DISCUSSION

The present findings highlight the limitations of light microscopy in accurately detecting mixed *Plasmodium* infections, particularly for *P. vivax*. Although all 500 samples were initially classified as mono-infections, molecular analyses revealed that approximately 20% were in fact mixed infections, demonstrating a substantial underestimation of mixed infections by microscopy. Notably, *P. vivax* was more frequently undetected than *P. falciparum*, suggesting a higher potential for silent circulation of this species. This finding is consistent with previous studies indicating that low-density *P. vivax* infections are easily missed by microscopy, especially in areas where *P. falciparum* prevalence is high [30, 31].

The underdiagnosis of mixed infections by microscopy is likely related to the low parasite density of the secondary infection, which frequently falls below the detection threshold of this method [31, 32]. This observation is consistent with our quantification assay results, which showed that *P. falciparum* generally exhibited higher parasite loads compared with *P. vivax* in both single and mixed infections. Consequently, microscopy tends to detect the predominant species, underestimating mixed infections and the true circulation of *P. vivax* in coendemic areas.

Temporal analysis further revealed distinct patterns of *P. vivax* underreporting. Between 2016 and 2018, the proportion of underreported cases decreased, followed by an increase, especially from 2020 onward. This trend correlated with the reported number of *P. falciparum* cases, suggesting that the likelihood of missing *P. vivax* infections may be influenced by the concurrent circulation of *P. falciparum*. Such patterns may have significant epidemiological implications, as undetected *P. vivax* infections may contribute to continued transmission, hampering elimination efforts.

The underreporting of *P. vivax* infections temporally coincided with the increasing frequency of *pvmdr1* gene amplification in Roraima. From 2020 onward, both the proportion of undetected *P. vivax* cases and the median copy number of *pvmdr1* progressively increased, with isolates carrying multiple copies becoming predominant (>70% in 2023–2024). This temporal convergence suggests that undetected infections may contribute to the persistence of parasite subpopulations carrying adaptive genetic traits, such as *pvmdr1* amplification, potentially influencing selection patterns. Chloroquine, the current first-line treatment for *P. vivax*, is unlikely to be the primary selective driver, as in vitro studies indicate that *pvmdr1* amplification is associated with lower CQ IC_50_ values in *P. vivax* [16]. In contrast, this gene amplification has been linked to reduced susceptibility to other antimalarials, particularly MQ.

Notably, studies on the orthologous *pfmdr1* gene in *P. falciparum* have demonstrated that increased copy number is associated with resistance to MQ and reduced sensitivity to artesunate (AS) [33]. In addition, gene duplications have been linked to decreased susceptibility to the structurally related arylamino alcohols MQ and lumefantrine (LUM), as well as to AS in Southeast Asia [34], and a higher prevalence of *pfmdr1* amplification has been reported in parasites collected after AL treatment in Africa [35]. Consistent with this, a recent efficacy trial in Angola confirmed that AL exerts strong selective pressure on *P. falciparum*, increasing the prevalence of *pfmdr1* amplification [26].

Here, we raise the possibility that drug pressure may be a driving force behind the emergence of gene amplifications in *P. vivax*, particularly in regions where *P. falciparum* and *P. vivax* cocirculate at high prevalence, as in the state of Roraima [10]. Although artesunate-MQ (AS-MQ) is not widely used as a second-line therapy for *P. falciparum* in Brazil, AL is the established first-line treatment [12], meaning that indirect exposure of *P. vivax* to LUM pressure cannot be excluded. In Brazil, mixed *P. falciparum*/*P. vivax* infections are treated with AL combined with PQ for 7 days to achieve a radical cure of the *P. vivax* malaria. Failure to detect mixed infections may therefore lead to inadequate radical cure, resulting in recurrent infections. As in *P. falciparum*, multiple copies of *mdr1* in *P. vivax* may potentially contribute to reduced LUM sensitivity, though this remains to be confirmed [26, 33]. Considering the intense cross-border movement between Roraima, Venezuela, and Guyana, parasite populations are likely exposed to similar therapeutic regimens across the region. In both Venezuela and Guyana, CQ combined with PQ remains the standard therapy for *P. vivax*, whereas AL is the first-line treatment for *P. falciparum* and mixed *P. vivax/P. falciparum* infections [36, 37].

Amplifications in the *pvmdr1* gene have been mainly described in Southeast Asia [25], likely due to the extensive use of MQ in treatment, whereas the overall prevalence in the Americas remains low [19]. An exception can be found in French Guiana, where studies have reported a high proportion of individuals carrying increased gene copy numbers, followed by a declining trend [18]. In our dataset, multiple copies of *pvmdr1* were found at a higher rate only in isolates from Roraima, where imported malaria plays an important role in the epidemiological scenario [38]. This region is part of the Guiana Shield, which includes French Guiana and where geographical proximity and the ease of cross-border impact malaria epidemiology in northern Brazil and neighboring countries [10].

In the specific context of Roraima, illegal gold mining represents an additional and important source of antimalarial drug pressure. In mining areas, the use of contraband antimalarial drugs, incomplete treatment courses, and unsupervised drug intake have been well documented [10, 39]. Such practices may result in subtherapeutic drug exposure, creating a selective environment distinct from that imposed by formal treatment guidelines, potentially influencing drug susceptibility in circulating parasite populations.

Importantly, according to the latest recommendations from the Brazilian Ministry of Health, the administration of AL for 3 days, combined with PQ for 14 days, is now indicated for recurrent *P. vivax* infections as an alternative to CQ-based regimens [12]. Furthermore, given the potential expansion of AS-MQ use to treat both *Plasmodium* species in Brazil, it is essential to investigate the potential impact of increased MQ and LUM exposure on *P. vivax* populations and the implications for treatment efficacy.

In addition to drug pressure, recent changes in malaria incidence in Roraima should be considered when interpreting the high frequency of *pvmdr1* amplification. The increase in malaria cases and the resurgence of *P. falciparum* may reflect shifts in parasite population dynamics that reshape genetic diversity, including the expansion of previously rare variants, potentially driven by stochastic processes such as genetic drift [40]. Therefore, the elevated *pvmdr1* copy number observed in Roraima may result from the combined effects of drug exposure and underlying demographic processes.

Our findings highlight the critical importance of ensuring accurate diagnosis and proper adherence to treatment. Without these measures, the inadequate use of antimalarials may generate unintended collateral effects, potentially contributing to the selection and spread of less susceptible *P. vivax* parasites, particularly in border regions. While our results strongly suggest an association between underdetection, drug pressure, and the rise of *pvmdr*1 amplification, they do not establish direct causality. Further longitudinal and functional studies are needed to confirm these relationships and clarify the mechanisms by which diagnostic limitations and treatment practices shape resistance dynamics in *P. vivax*.

## Supplementary Material

jiag196_Supplementary_Data

## Supplementary Data

Supplementary materials are available at *The Journal of Infectious Diseases* online (http://jid.oxfordjournals.org/). Supplementary materials consist of data provided by the author that are published to benefit the reader. The posted materials are not copyedited. The contents of all supplementary data are the sole responsibility of the authors. Questions or messages regarding errors should be addressed to the author.
