# Supplementary material for: Hidden Infections, Emerging Resistance: Underdiagnosed Plasmodium vivax and Multidrug Resistance-1 (pvmdr1) Gene Amplification in the Guiana Shield
Source: J Infect Dis. 2026 Apr 8;233(6):e1480–9. doi: 10.1093/infdis/jiag196 (PMC13271397; doi:10.1093/infdis/jiag196)
Supplement: jiag196_Supplementary_Data [file jiag196_supplementary_data.docx]

**SUPPLEMENTARY MATERIAL**

*Supplement to*: **Hidden Infections, Emerging Resistance: Underdiagnosed *Plasmodium vivax* and multidrug resistance-1 (*pvmdr1*) gene Amplification in the Guiana Shield**

Maria Carolina Silva de Barros Puça, Maria Eduarda Pereira Mascarenhas, Queralt Bullich Huguet, Yanka Evellyn Alves Rodrigues Salazar, Iuri Rodrigues Nogueira, Jaime Louzada_,_ Anielle de Pina-Costa, André Machado de Siqueira, Michelle de Oliveira e Silva, Dhélio Batista Pereira, Simone Ladeia-Andrade, Marcelo Urbano Ferreira, Joseli de Oliveira Ferreira, José Pedro Gil, Tais Nobrega de Sousa

**METHODS**

**Study site and population**

**Study Area**

For this study, we evaluated samples from three epidemiologically and geographically distinct areas in Brazil.

Roraima is in the northernmost part of Brazil, near the borders with Venezuela and Guyana. Its geographical location, with easy road access to both countries via highways BR-174 and BR-401, respectively, facilitates the importation and spread of malaria cases. Boa Vista, the state capital and main urban center, concentrates most malaria diagnoses and treatments, serving both local residents and migrants. In contrast, Pacaraima, located 231 km north of Boa Vista along BR-174 and bordering Santa Elena de Uairén (Venezuela), represents a major entry point for imported infections, particularly linked to intense population mobility and mining-related activities [1,2]. Although malaria incidence has been declining in most of the Amazon region, a different trend has been observed in areas like Roraima, where the number of imported malaria cases has increased since 2017 [2]. As a result, this state has become the Brazilian state with the highest number of imported cases from neighboring countries. Despite being considered a low-transmission area, concerning epidemiological data have emerged in recent years [1].

Between 2016 and 2024, a total of 56,679 malaria cases were recorded in Boa Vista through the Brazilian Malaria Surveillance System (SIVEP-Malaria). Malaria cases in this city showed varying trends according to the infecting species. *Plasmodium vivax* consistently accounted for the majority of cases, ranging from 1,724 in 2016 to a peak of 8,432 in 2022, before declining to 2,460 in 2024. *Plasmodium falciparum* infections remained less frequent, with the highest number reported in 2022 (3,260 cases). Mixed infections by *P. vivax* and *P. falciparum* were relatively uncommon throughout the period, ranging from 78 cases in 2016 to 898 in 2022, representing a smaller fraction of total malaria cases each year. Overall, the data show a predominance of *P. vivax* (76%) across the study period, with fluctuating but lower counts of *P. falciparum* (20%) and mixed infections (4%).

Rondônia is in the western part of the Brazilian Amazon. Unlike Roraima, where intense cross-border migration has been associated with a high proportion of imported malaria cases[1,3]. There is no evidence indicating a similar pattern in Rondônia. Porto Velho is primarily affected by internal migration related to regional economic activities, including agriculture, logging, and infrastructure development. Although malaria transmission persists in Rondônia, its dynamics are primarily driven by local factors rather than importation. As a result, this state experiences a more stable but endemic malaria scenario, with fewer fluctuations caused by imported cases. In 2023, a total of 7,811 malaria cases were reported in Porto Velho, of which 94% were caused by P. vivax, 5.4% by P. falciparum, and only 0.5% were mixed infections, according to the Brazilian Malaria Surveillance System (SIVEP-Malaria).

The third set of samples was collected from Acre, located in the northwest of Brazil in the Juruá Valley, near the border with Peru. While malaria transmission in Brazil typically occurs in rural areas, only about 17% of cases occur in urban zones; this proportion rises to 45% in the urban area of Mâncio Lima/Acre [4,5]. In Mâncio Lima, a total of 6,302 malaria cases were reported in 2014, of which 76.2% were caused by P. vivax, 23.3% by P. falciparum, and 0.5% were mixed infections. In 2015, 5,593 cases were recorded, with P. vivax accounting for 84.9%, P. falciparum for 14.8%, and mixed infections for 0.3% (SIVEP-Malaria).

**Study Population**

In Roraima, patients older than 12 years presenting with symptomatic malaria were recruited at the Cosme e Silva Polyclinic, one of the main malaria diagnostic and treatment centers in Boa Vista, which accounts for nearly one-third of all reported cases in the city, highlighting its central role in regional surveillance [6], between 2016 and 2024. Recruitment was conducted in three phases: from 2016 to 2018 as part of [2]; from 2019 to 2020 within the framework of [7]; and from 2023 to 2024 as part of the ABRACAMAL project (A Multicenter Brazilian Amazon Cohort for Malaria, Gates Foundation grant INV-003970). Both *P. vivax* and *P. falciparum* infections were included, confirmed by thick blood smear following Brazilian Ministry of Health guidelines. Each patient’s malaria history was retrieved from the official Malaria Epidemiological Surveillance System (SIVEP-Malaria).

Treatment followed national guidelines: chloroquine (25 mg/kg over 3 days) plus primaquine (3.5 mg/kg over 7 days) for *P. vivax*, and artemether–lumefantrine (AL) as the first-line artemisinin-based combination therapy (ACT) for *P. falciparum*, combined with a single primaquine dose (0.75 mg/kg) to clear gametocytes [8]. Individuals with contraindications to primaquine, severe disease, pregnancy, or recent antimalarial use were not eligible for inclusion.

Patients from the Acre study were males and females aged 5–70 years living in urban and peri-urban areas of Mâncio Lima. Between June 2014 and July 2015, all patients were treated at one of the three government-run malaria clinics in the municipality. Each case was confirmed as a *P. vivax* mono-infection by both microscopy and PCR. Molecular confirmation was performed using venous blood samples. No minimum parasitemia threshold was required for inclusion. Exclusion criteria included recent use of antimalarials (within the previous two weeks), hypersensitivity or allergy to CQ and/or PQ, complicated or severe malaria, severe anemia (hemoglobin < 8.0 g/100 mL), G6PD deficiency, pregnancy or breastfeeding, severe malnutrition (weight-for-age z-score ≤ –3), and history of serious chronic conditions (e.g., cardiovascular or psychiatric disorders, HIV/AIDS, chronic kidney failure, liver cirrhosis) [9]. Between June 2014 and July 2015, as part of a previous study, half of the samples were treated with CQ and the other half with CQ+PQ. Of the 204 samples originally collected, 197 were available for this study. Among them, all 38 samples from patients who experienced relapse were selected, along with a comparison group of 54 samples from patients without relapse during the study period.

In Porto Velho, Rondônia, patients with P. vivax infection were enrolled at CEPEM between March and September 2023 as part of the ABRACAMAL project. Both males and females were included, with ages ranging from 18 to 69 years. Diagnosis was initially established by thick blood smear, and parasitemia levels were quantified per leukocyte count. Molecular confirmation of P. vivax infection was additionally performed by qPCR at CEPEM and Fiocruz-Minas. Treatment followed national guidelines, comprising either chloroquine plus primaquine or artemether-lumefantrine plus primaquine, depending on clinical evaluation. Recurrence data were systematically collected, and some patients presented at least one documented recurrence within the follow-up period.

**Malaria molecular diagnosis**

DNA was extracted from 200 µL of whole blood samples using the QIAamp DNA Mini Kit (QIAGEN, Minneapolis, MN, USA), following the manufacturer’s instructions. All reagents, except absolute ethanol, were provided by the manufacturer, including purification columns and collection tubes. Extracted DNA was finally eluted in 80 µL of the provided elution buffer.

For Roraima samples, the quantitative real-time PCR (qPCR) protocol used for molecular diagnosis of *Plasmodium spp*. was based on the amplification of Pfr364 and Pvr47 for the detection of *P. falciparum* and *P. vivax*, respectively, as previously described [10]. For *P. vivax*, the reagents and cycling conditions used were as follows: primers F (5′-TCCGCAGCTCACAAATGTTC-3′) and R (5′-ACATGGGGATTCTAAGCCAATTTA-3′) at final concentrations of 0.05 μM and 0.9 μM, respectively, probe (6FAM 5′-TCCGCGAGGGCTGCAA-3′ BHQ1) at 0.25 μM final concentration, 5 μL of TaqMan Universal Master Mix 2× (Thermo Fisher Scientific), 2 μL of extracted DNA (~50 ng), and water for a final volume of 10 μL. For *P. falciparum*, primers F (5′-CTCGCAATAACGCTGCAT-3′) and R (5′-TTCCCTGCCCAAAAACG-3′) had final concentrations of 0.9 μM and 0.3 μM, respectively, probe (6FAM 5′-TGGTGCCGGGGGTTTCTACGC-3′ BHQ1) at 0.15 μM final concentration, 5 μL of TaqMan Universal Master Mix 2× (Thermo Fisher Scientific), 2 μL of extracted DNA (~50 ng), and water for a final volume of 10 μL.

For Acre samples, the molecular diagnosis of malaria was performed by quantitative real-time PCR targeting a species-specific 100-bp fragment of the P. falciparum and P. vivax 18S rRNA genes, as previously described [11]. Amplification was carried out on a Step One Plus Real-Time PCR System (Applied Biosystems, Foster City, CA). The detection threshold of this diagnostic PCR is approximately 3 parasites/μl of blood. No-template controls, containing all reagents for amplification except the DNA template, were included in each PCR microplate. Parasite density estimates obtained by PCR and expert microscopy were strongly correlated (Spearman correlation coefficient [rs] = 0.675; P < 0.0001).

**Determination of Copy Number Variation of the *pvmdr1* Gene**

The copy number variation of the pvmdr1 gene was determined by quantitative real-time PCR (qPCR) using previously described hydrolysis probes and primers, with the P. vivax β-tubulin gene used as the reference gene [12,13].

Amplification reactions were performed in a final volume of 10 μL containing 5 μL of Taqman® Universal PCR Master Mix 2× (Thermo Fisher Scientific), 900 nM of forward primer for *pvmdr1* or 300 nM for *pvtubulin*, 900 nM of reverse primer for both genes, 200 nM probe for *pvmdr1* or 250 nM for *pvtubulin,* and 1 μL of DNA (~10 ng/μL). The cycling parameters were as follows: initial denaturation at 95°C for 10 minutes, followed by 40 cycles of 15 seconds at 95°C and 1 minute at 60°C. PCR was run in triplicate on a CFX Opus 96 (Bio-Rad) using 96-well plates.

Control samples with a single copy and duplication of the target gene were used as calibrators. The ΔΔCt method was applied to estimate the copy number of the *pvmdr1* gene relative to *pvtubulin*. A calibration curve was created using plasmids containing the *pvmdr1* and *pvtubulin* inserts; one plasmid contained one copy of *pvmdr1* and one copy of *pvtubulin,* and another contained two copies of *pvmdr1* and one copy of *pvtubulin*. Samples were considered to have one copy when the relative quantification value was between 0.5 and 1.4, and samples with a minimum relative quantification ≥1.4 were defined as amplified.

Only samples with a cycle threshold (Ct) <35 and a Ct standard deviation <0.5 were included in the analysis. Each experiment was performed in triplicate, and gene amplification was confirmed in at least two independent experiments.

**SUPPLEMENTARY TABLES**

**Supplementary Table 1.** Copy number variation in *pvmdr1* in *Plasmodium vivax* isolates from Brazilian Amazon states.

| **Brazilian State** | **Year^a^** | **N^b^** | **Gene Copy Number** | | **Reference** |
| --- | --- | --- | --- | --- | --- |
|  |  |  | **1 (n)** | **≥ 2 (n)** |  |
| Acre | 2004 - 2011 | 215 | 99% (213) | 1% (2) | [14] |
| Acre | 2014 - 2015 | 84 | 100% (84) | 0% (0) | Data from this study |
| Amazonas | 2012 - 2013 | 37 | 88% (33) | 12% (4) | [15] |
| Mato Grosso | 2002 – 2012 | 13 | 54% (7) | 46% (6) | [13] |
| Roraima | 2016 - 2024 | 211 | 40% (84) | 60% (127) | Data from this study |
| Rondônia | 2008 | 22 | 96% (21) | 4% (1) | [13] |
| Rondônia | 2023 | 105 | 99% (104) | 1% (1) | Data from this study |

^a^ Year of sample collection.

^b^ Number of isolates.

**Supplementary Table 2.** Number and proportion (%) of *Plasmodium vivax* and *P. falciparum* mono-infections detected by microscopy among samples included in the study, by year (2016–2024). Infections underreported by routine microscopy were identified through molecular analysis. The “Total, N” column represents the total number of mono-infections analyzed per year.

| **Year** | **Mono-infections by Microscopy, n (%) [IC95%]** | | **Total, N** | **Underreporting by qPCR, n (%) [IC95%]** | | **Total, N** |
| --- | --- | --- | --- | --- | --- | --- |
|  | ***P. vivax*** | ***P. falciparum*** |  | ***P. vivax*** | ***P. falciparum*** |  |
| 2016 | 24 (60) [44.6-73.7] | 16 (40) [26.3-55.3] | 40 | 5 (13) [5.5-26.1] | 2 (5) [1.4-16.5] | 7 |
| 2017 | 47 (53) [42.5-62.8] | 42 (47) [37.2-57.5] | 89 | 9 (10) [5.4-18.1] | 1 (1) [0.2-6.1] | 10 |
| 2018 | 28 (57) [43.3-70] | 21 (43) [30.0-56.7] | 49 | 3 (6) [2.1-16.5] | 0 (0) [0.0-7.3] | 3 |
| 2019 | 24 (63) [47.3-76.6] | 14 (37) [23.4-52.7] | 38 | 0 (0) [0.0-9.2] | 0 (0) [0.0-9.2] | 0 |
| 2020 | 43 (74) [61.6-83.7] | 15 (26) [16.3-38.4] | 58 | 3 (5) [1.8-14.1] | 0 (0) [0.0-6.2] | 3 |
| 2022* | 0 (0) [0.0-8.0] | 44 (100) [92.0-100] | 44 | 31 (70) [55.8-81.8] | NA | 31 |
| 2023 | 85 (70) [61.0-77.1] | 37 (30) [22.9-39.9] | 122 | 8 (7) [3.4-12.4] | 20 (16) [10.9-24.0] | 28 |
| 2024 | 36 (60) [47.4-71.4] | 24 (40) [28.6-52.6] | 60 | 10 (17) [9.3-28.0] | 6 (10) [4.7-20.1] | 16 |
| **Total** | 287 (57) | 213 (43) | 500 | 69 (70) | 29 (30) | 98 |

*Included only in the overall analysis due to the absence of corresponding *P. vivax* samples.

**Supplementary Table 3.** Cases of *Plasmodium vivax*, *P. falciparum*, and *P. vivax/P. falciparum* mixed infections reported through the SIVEP-Malaria system in Boa Vista, Roraima, Brazil (2016–2024).

| **Year** | **Reported Cases, N (%)** | | | **Total^a^** |
| --- | --- | --- | --- | --- |
|  | ***P. falciparum*** | ***P. vivax*** | **Mixed** |  |
| 2016 | 567 (23.9%) | 1724 (72.8%) | 78 (3.3%) | 2369 |
| 2017 | 590 (16.1%) | 2979 (81.2%) | 102 (2.8%) | 3671 |
| 2018 | 732 (12.8%) | 4885 (85.5%) | 96 (1.7%) | 5713 |
| 2019 | 590 (9.1%) | 5779 (89.4%) | 96 (1.5%) | 6465 |
| 2020 | 1356 (20.8%) | 4949 (75.7%) | 229 (3.5%) | 6534 |
| 2021 | 1541 (20.0%) | 5898 (76.5%) | 272 (3.5%) | 7711 |
| 2022 | 3260 (25.9%) | 8432 (67.0%) | 898 (7.1%) | 12590 |
| 2023 | 1918 (23.6%) | 5727 (70.4%) | 492 (6.0%) | 8137 |
| 2024 | 840 (24.1%) | 2460 (70.5%) | 189 (5.4%) | 3489 |

Data were obtained from the SIVEP system.

^a^ Total number of reported malaria cases per year.

**Supplementary Table 4.** Geometric means comparisons of parasite densities for *P. falciparum* and *P. vivax* in both single and mixed infections.

| **Microscopy** | **qPCR quantification (parasites/μL)** | | **Total^a^** | ***P*-value^b^** |
| --- | --- | --- | --- | --- |
|  | ***P. falciparum***  **GeoMean (95% CI)** | ***P. vivax***  **GeoMean (95% CI)** |  |  |
| *P. falciparum* | 129,462 (75,418 - 222,232) | 43.3 (9.1 - 97.9) | 51 | <0.0001 |
| *P. vivax* | 466.9 (87.5 - 2,489) | 50,324 (24,690 - 102,568) | 26 | <0.0003 |
| Mixed | 20,739 (5,901 - 72,879) | 1,633 (293.2 - 9,094) | 13 | 0.0134 |

GeoMean, Geometric mean (parasites/µL); CI, confidence interval

^a^ Total number of samples analyzed per infection group.

^b^ *P*-value calculated using the Wilcoxon rank-sum test.

**REFERENCES**

1. Abdallah R, Louzada J, Carlson C, et al. Cross-border malaria in the triple border region between Brazil, Venezuela and Guyana. Sci Rep. **2022**; 12(1):1200.

2. Louzada J, Almeida NCV de, Araujo JLP de, et al. The impact of imported malaria by gold miners in Roraima: characterizing the spatial dynamics of autochthonous and imported malaria in an urban region of Boa Vista. Mem Inst Oswaldo Cruz. **2020**; 115:e200043.

3. Albuquerque NRM de, Corder RM, Johansen IC, et al. The source-sink dynamics of Plasmodium vivax may undermine malaria elimination efforts in the Amazon: an epidemiological and population genomic study. J Infect Dis. **2025**; 4:jiaf457.

4. Salla LC, Rodrigues PT, Corder RM, Johansen IC, Ladeia-Andrade S, Ferreira MU. Molecular evidence of sustained urban malaria transmission in Amazonian Brazil, 2014–2015. Epidemiol Infect. **2020**; 148:e47.

5. Johansen IC, Rodrigues PT, Ferreira MU. Human mobility and urban malaria risk in the main transmission hotspot of Amazonian Brazil. PLoS One. **2020**; 15(11):e0242357.

6. Barros J de A, Granja F, Silva D da S e, Citó AC, Peterka C, Ferreira-da-Cruz M de F. A snapshot of a representative Brazilian state of illegal mining in indigenous areas during the era of malaria elimination. Cad Saude Publica. **2024**; 40(6):e00224023.

7. Salazar YEAR, Louzada J, Puça MCS de B, et al. Delayed gametocyte clearance in Plasmodium vivax malaria is associated with polymorphisms in the cytochrome P450 reductase (CPR). Antimicrob Agents Chemother. **2024**; 68(4):e0120423.

8. Ministerio da Saude. Guia de tratamento da malária no Brasil. Ministério da Saúde; 2021. Available from: www.bvsms.saude.gov.br.

9. Ladeia-Andrade S, Menezes MJ, Sousa TN de, et al. Monitoring the Efficacy of Chloroquine-Primaquine Therapy for Uncomplicated Plasmodium vivax Malaria in the Main Transmission Hot Spot of Brazil. Antimicrob Agents Chemother. **2019**; 63(5):e01965.

10. Amaral LC, Robortella DR, Guimarães LFF, et al. Ribosomal and non-ribosomal PCR targets for the detection of low-density and mixed malaria infections. Malar J. **2019**; 18(1):154.

11. Ladeia-Andrade S, Melo GNP de, Souza-Lima R de C de, et al. No Clinical or Molecular Evidence of Plasmodium falciparum Resistance to Artesunate–Mefloquine in Northwestern Brazil. The American Society of Tropical Medicine and Hygiene. **2016**; 95(1):148–154.

12. Imwong M, Pukrittayakamee S, Pongtavornpinyo W, et al. Gene Amplification of the Multidrug Resistance 1 Gene of *Plasmodium vivax* Isolates from Thailand, Laos, and Myanmar. Antimicrob Agents Chemother. **2008**; 52(7):2657–2659.

13. Costa GL, Amaral LC, Fontes CJF, Carvalho LH, Brito CFA de, Sousa TN de. Assessment of copy number variation in genes related to drug resistance in Plasmodium vivax and Plasmodium falciparum isolates from the Brazilian Amazon and a systematic review of the literature. Malar J. **2017**; 16(1):152.

14. Vargas-Rodríguez R del CM, Silva Bastos M da, Menezes MJ, Orjuela-Sánchez P, Ferreira MU. Single-Nucleotide Polymorphism and Copy Number Variation of the Multidrug Resistance-1 Locus of Plasmodium vivax: Local and Global Patterns. The American Society of Tropical Medicine and Hygiene. **2012**; 87(5):813–821.

15. Silva SR, Almeida ACG, Silva GAV da, et al. Chloroquine resistance is associated to multi-copy pvcrt-o gene in Plasmodium vivax malaria in the Brazilian Amazon. Malar J. **2018**; 17(1):267.

# 
